# Generation of Adducts of 4-Hydroxy-2-nonenal with Heat Shock 60 kDa Protein 1 in Human Promyelocytic HL-60 and Monocytic THP-1 Cell Lines

**DOI:** 10.1155/2015/296146

**Published:** 2015-05-20

**Authors:** Alessia Arcaro, Martina Daga, Giovanni Paolo Cetrangolo, Eric Stefano Ciamporcero, Alessio Lepore, Stefania Pizzimenti, Claudia Petrella, Maria Graf, Koji Uchida, Gianfranco Mamone, Pasquale Ferranti, Paul R. J. Ames, Giuseppe Palumbo, Giuseppina Barrera, Fabrizio Gentile

**Affiliations:** ^1^Dipartimento di Medicina e Scienze della Salute, Università del Molise, 86100 Campobasso, Italy; ^2^Dipartimento di Scienze Cliniche e Biologiche, Università di Torino, 10125 Torino, Italy; ^3^Dipartimento di Medicina Molecolare e Biotecnologie Mediche, Università di Napoli Federico II, 80131 Napoli, Italy; ^4^Graduate School of Bioagricultural Science, Nagoya University, Nagoya 464-8601, Japan; ^5^Centro di Spettrometria di Massa Proteomica e Biomolecolare, ISA-CNR, 83100 Avellino, Italy; ^6^Dipartimento di Agraria, Università di Napoli “Federico II”, Portici, 80055 Napoli, Italy; ^7^William Harvey Research Institute, Queen Mary University London, London E1 4NS, UK

## Abstract

Heat shock 60 kDa protein 1 (HSP60) is a chaperone and stress response protein responsible for protein folding and delivery of endogenous peptides to antigen-presenting cells and also a target of autoimmunity implicated in the pathogenesis of atherosclerosis. By two-dimensional electrophoresis and mass spectrometry, we found that exposure of human promyelocytic HL-60 cells to a nontoxic concentration (10 *μ*M) of 4-hydroxy-2-nonenal (HNE) yielded a HSP60 modified with HNE. We also detected adducts of HNE with putative uncharacterized protein CXorf49, the product of an open reading frame identified in various cell and tissue proteomes. Moreover, exposure of human monocytic THP-1 cells differentiated with phorbol 12-myristate 13-acetate to 10 *μ*M HNE, and to light density lipoprotein modified with HNE (HNE-LDL) or by copper-catalyzed oxidation (oxLDL), but not to native LDL, stimulated the formation of HNE adducts with HSP60, as detected by immunoprecipitation and western blot, well over basal levels. The identification of HNE-HSP60 adducts outlines a framework of mutually reinforcing interactions between endothelial cell stressors, like oxLDL and HSP60, whose possible outcomes, such as the amplification of endothelial dysfunction, the spreading of lipoxidative damage to other proteins, such as CXorf49, the activation of antigen-presenting cells, and the breaking of tolerance to HSP60 are discussed.

## 1. Introduction

The decomposition of lipid peroxides during oxidative stress releases a number of reactive aldehydic intermediates (ketoaldehydes, 2-alkenals, and 4-hydroxy-2-alkenals) [1, 2] that may induce protein modifications. The most reactive compound is 4-hydroxy-2-nonenal (HNE) [3, 4] that plays a significant role in the pathogenesis of a wide spectrum of human diseases ranging from chronic inflammatory and autoimmune diseases to neurodegeneration and cancer [5, 6]. By a redox proteomics approach, several functional groups of cell proteins of critical importance for cell metabolism and survival appeared to be exposed more than others to the adverse consequences of redox imbalance and hence HNE under cell stressful conditions [7, 8]. To clarify the molecular basis of HNE antiproliferative effects previously observed in the macrophage-derived, human leukemic HL-60 cell line [9], we searched previously for HNE-modified proteins in the proteome of these cells. After 15-minute exposure to a nontoxic dose (10 *μ*M) of HNE, we detected HNE adducts with *α*-enolase in close vicinity of the plasmamembrane [10]. In the present study, under identical conditions but for longer time exposure (2 hours), we extended our search to protein targets of HNE in HL-60 cells and identified HNE adducts with heat shock 60 kDa protein 1 (HSP60) and putative uncharacterized protein CXorf49 (UniProt identifier: A8MYA2 - CX049_HUMAN), the product of an open reading frame on chromosome X [11] (HGNC [Human Gene Nomenclature Committee] identifier: 30891) recently also detected in proteomic analyses of various cell types and tissues [12–14].

Human HSP60 is a member of a superfamily of highly conserved HSPs that controls protein folding and stress responses [15]. By associating with endogenous cell peptides and binding to scavenger receptors, such as the lectin-like oxidized LDL receptor-1 (LOX-1) at the surface of dendritic cells (DCs) and macrophages, HSPs are able to convey cell antigens into the presentation machinery of antigen-presenting cells (APCs) [16]. Furthermore, HSP60 and other HSPs can function as potent danger-associated molecular patterns (DAMPs) [17, 18].

In addition to HSP60, LOX-1 also recognizes proatherogenic oxidized LDL (oxLDL) that is strongly implicated in the initiation and progression of human atherosclerosis [19, 20]. HNE-histidine adducts served as LOX-1 ligands in oxLDL and HNE-modified proteins and strongly contributed [21] to a number of cell dysfunctions determined by oxLDL in endothelial cells (ECs) and macrophages [22–30]. Upon binding to LOX-1 on DCs, oxLDL were also able to induce DC maturation [31]. HSP60 itself is recognized as a prominent target of autoimmune responses in atherosclerosis. The role of HSP60 as an immunopathogenic self-antigen in atherosclerosis is supported by several reports of HSP60-specific autoreactive T cells in atherosclerotic patients [32–35]. Therefore, we next set to investigate, by an immunoprecipitation and western blotting strategy, whether HNE in protein-bound form, namely, HNE bound to LDL, might incite the production of endogenous HNE and the formation of adducts of HNE with cell proteins, particularly HSP60, in the human monocytic leukemic THP-1 cell line. In fact, after differentiation induced with phorbol 12-myristate 13-acetate (PMA), these cells have been indicated as a useful experimental model of the interactions of human macrophages with vascular cells within the context of atherogenesis [36]. Upon exposure to HNE, HNE-LDL, and LDL oxidized with CuSO_4_, but not to native LDL, HNE-HSP60 adducts were formed in THP-1 cells at levels well higher than in basal conditions.

The interest of finding HNE-HSP60 adducts in HL-60 cells exposed to HNE is severalfold. The formation of HNE-HSP60 adducts might represent the modification of self which is required for the breaking of tolerance to HSP60. HNE modification might confer upon HSP60 the properties of an EC stressor and adjuvant, capable of cooperating in the promotion of endothelial dysfunction and APC differentiation, and of a modified self-antigen, both of which may favor the transition from the inflammatory reaction to the products of lipid peroxidation and their adducts with cell proteins to an autoimmune adaptive response on the way to atherosclerosis.

## 2. Materials and Methods

### 2.1. Cells and Culture Conditions

HL-60 and THP-1 cells were cultured at 37°C in humidified atmosphere of 5% CO_2_ in air, in RPMI 1640 medium supplemented with 0.002 M glutamine, antibiotics, and 10% heat-inactivated foetal bovine serum (FBS) (Biochrom AG Seromed). Cells were monitored daily for growth rate and cell viability by the trypan blue exclusion test (Sigma-Aldrich).

### 2.2. Isolation and Modification of LDL

LDL was prepared from EDTA-plasma of a normolipemic, fasting (12–14 h), male healthy donor, by two short runs of zonal ultracentrifugation in KBr in the density range of 1.006–1.225 g/mL. This procedure significantly shortens the isolation time (3.5 h), thereby minimizing the spontaneous oxidation of LDL [37]. All isolation and successive modification steps with LDL were performed under sterile conditions. Protection of LDL from the action of proteases and phosphatases was ensured by addition of 2 × 10^−6^ M aprotinin and 1 mM EDTA. After isolation, LDL was transferred to PBS, 1 mM EDTA by solvent exchange on 10 mL Sephadex G 25 PD-10 gel filtration columns (GE Healthcare) and further sterilized by filtration through sterile 0.22 *μ*m pore-size microfilters. Before usage, whenever required, LDL was transferred to EDTA-free PBS by the same technique. LDL concentration was assayed by a copper-chelate method. Native LDL was kept in air-tight vials with no unused residual space and was used as soon as possible after isolation. HNE modification of LDL at the concentration of 0.9 mg/mL was performed by reaction with 1 mM HNE in PBS at 37°C for 20 h. Metal-catalyzed oxidation was performed by incubation with 20 *μ*M CuSO_4_ in PBS at 37°C for 20 h.

### 2.3. Cell Treatments

Before treatment with HNE, HL-60 cells were washed and resuspended in serum-free medium. 4-Hydroxy-2-nonenal (HNE) (Calbiochem) was added to the cell suspension (2 × 10^5^ cells/mL) at the final concentration of 10 *μ*M. After 2 h from the start of the treatment, cell viability was controlled with the trypan blue dye exclusion test and 10 × 10^6^ cells were harvested, washed twice in cold PBS, pH 7.4, and used for protein extraction.

Before exposing THP-1 cells to HNE, native or modified LDL, their macrophagic differentiation was induced with phorbol 12-myristate 13-acetate (PMA). Cells were passaged, while in the exponential growth phase, at the density of 1 × 10^6^ cells/mL, in 60 mm Petri dishes, were cultured in RPMI-1640 with 10% FBS for 24 h and then incubated with 30 ng/mL PMA for 48 h. Before the addition of stimuli, cells were maintained in serum-free medium for another 10 h.

### 2.4. Detection of Reactive Oxygen Species (ROS)

The detection of reactive oxygen species (ROS) was performed fluorimetrically. Briefly, 2 h after the addition of 10 *μ*M HNE, 5 × 10^6^ HL-60 cells were harvested, centrifuged, resuspended in 40 *μ*L of PBS, and lysed by sonication. To the cell lysate, 10 *μ*L of 5 *μ*M 2′-7′-dichlorofluorescein diacetate (DCFH-DA) was added. The mixture was incubated at 37°C for 20 min. Under these conditions, DCFH-DA was hydrolysed to nonfluorescent DCFH, which was rapidly oxidized to DCF (2′-7′-dichlorofluorescein) in the presence of ROS. The fluorescence generated by the oxidation of DCFH was measured and used for the quantification of ROS. The reaction was stopped on ice by adding 1 mL of 0.1 M phosphate buffer, pH 7.4, containing 0.1% Triton X-100.

### 2.5. Antibodies

The mouse anti-HNE-histidine monoclonal antibody used in this study was prepared in-house by Dr. Koji Uchida. Polyclonal anti-HSP60 antibodies used included H-300 rabbit anti-HSP60 antibodies (sc-13966, Santa Cruz Biotechnology), raised against amino acids 274–573 at the C terminus of human HSP60, and N-20 goat anti-HSP60 antibodies (sc-1052, Santa Cruz Biotechnology), raised against a N-terminal peptide of HSP60 of human origin.

### 2.6. Confocal and Epifluorescence Microscopy

5 × 10^4^ HL-60 cells were smeared onto a noncharged slide, fixed for 15 min in 4% paraformaldehyde, washed twice, permeabilized with 1% Triton X-100 at rt for 30 min, and washed with PBS, pH 7.4. Thereafter, the slides were incubated with 1% BSA in PBS, pH 7.4, at rt for 30 min, and then with the anti-HNE-histidine primary antibody at 4°C overnight. The slides were washed, incubated at rt for 1 h with secondary fluorochrome-conjugated antibodies, washed again, mounted on coverslips, and examined with a Leica TCS SP2 confocal microscope, equipped with a HCX Apo 0.8 water immersion objective. For epifluorescence microscopic analysis (Axioskop, Carl Zeiss), slides were also counterstained with 4′,6-diamidino-2-phenylindole (DAPI).

### 2.7. Electron Microscopy

HL-60 cells were fixed and samples were infiltrated in 2 : 1 (v : v) ethanol : LRW (London Resin White) for 1 h, 1 : 2 (v : v) ethanol : LRW for 2 h and 100% LRW overnight at 4°C; all other steps were carried out at rt. Samples were embedded in gelatin capsules [38] and processed for ultramicrotomy [39]. Single-antibody immunogold localization was performed as previously described [40].

### 2.8. Preparation of HL-60 and THP-1 Cell Protein Extracts

Total cell protein extracts of HL-60 cells exposed to HNE were prepared by suspending 10 × 10^6^ cells in lysis buffer, containing 0.05 M Tris/HCl, pH 7.4, 0.15 M NaCl, 0.005 M EDTA, 1% Nonidet, 0.001 M PMSF, 0.001 M sodium orthovanadate, and 0.05% aprotinin. Total cell protein extracts of THP-1 cells differentiated with PMA and exposed to HNE or native or modified LDL were prepared by scraping the cells off Petri dishes and suspending them in lysis buffer, containing 0.02 M Tris/HCl, pH 7.4, 0.15 M NaCl, 1% Triton X-100, 10% glycerol, plus protease inhibitor, and phophatase inhibitor cocktails (Roche). Debris was discarded by centrifugation at 13000 g at 4°C. Protein concentration was assayed by a dye-binding technique (Bio-Rad Laboratories).

### 2.9. Two-Dimensional Polyacrylamide Gel Electrophoresis (2-DE)

Proteins in HL-60 cell lysates were separated by two-dimensional gel electrophoresis (2-DE), using isoelectrofocusing (IEF) in immobilized pH 3–10 gradients, followed by SDS-PAGE in 8–18% total acrylamide gradient gels. Separate cell lysates obtained under test and control conditions were analyzed simultaneously, and two replicate separations were performed with each cell lysate, one destined for silver staining (see Supplemental Materials and Methods in Supplementary Material available online at http://dx.doi.org/10.1155/2015/296146) and the other for immunoblotting with the anti-HNE-histidine antibody. Repeated separations were performed per each experimental condition. Analytical separations were performed with cell lysate aliquots containing 50–200 *μ*g of total proteins each. Detailed information can be found in Supplemental Materials and Methods.

### 2.10. Electrophoretic Transfer and Immunodetection of Cell Proteins Separated in 2-DE Gels

Cell proteins separated by 2-DE were transferred onto polyvinylidene difluoride (PVDF) membranes (Immobilon P, Millipore) by semidry electrophoretic transfer in 0.025 M Tris base, 0.01 M glycine, at the constant current of 0.8 mAmp/cm^2^ for 1.5 h, using a Multiphor II apparatus equipped with Novablot graphite electrode plates (GE Healthcare). At the end of the transfer, nonspecific binding sites on PVDF membranes were blocked with 5% blotting-grade nonfat dry milk (NDM) (Bio-Rad Laboratories) in PBS, pH 7.4, and 0.2% Tween 20, at 4°C overnight. Membranes were rinsed 3 times in PBS, pH 7.4, 0.2% Tween 20, and incubated with a murine monoclonal anti-HNE-histidine primary antibody (prepared in-house by Dr. Koji Uchida), at the dilution of 1 to 2000 in 5% NDM in PBS, pH 7.4, and 0.2% Tween 20, at rt for 1 h. Membranes were washed twice in PBS, pH 7.4, and 0.2% Tween 20 for 15 min each time and incubated with HRP-conjugated, secondary anti-murine IgG antibodies, at the dilution of 1 : 5000 in 2.5% NDM in PBS, pH 7.4, and 0.2% Tween 20, at rt for 1 h. Optimal dilutions of antibodies, as judged from the signal-to-noise ratio, were selected in test experiments conducted with various dilutions of primary (from 1 : 800 to 1 : 6400) and secondary antibody (from 1 : 8000 to 1 : 64000). The Tween 20 concentration was also optimized. Finally, membranes were washed twice in PBS-Tween for 15 min each time, incubated in ECL Plus developer (GE Healthcare) at rt for 5 min and chemiluminescence detected with Hyperfilm ECL (GE Healthcare).

### 2.11. Identification of HNE-Modified Proteins by Mass Spectrometry

Standard patterns of 2-DE separation of the proteome of HL-60 cells, under basal conditions and after exposure to HNE, were derived from computer-assisted overlay of the scans of multiple replicate, analytical 2-DE gels, stained with silver nitrate or probed with anti-HNE-histidine antibodies. Bidimensional maps of silver-stained proteins were aligned with the corresponding chemiluminescent detection maps, in order to locate the proteins detected by anti-HNE-histidine antibodies within the bidimensional separation pattern. Moreover, separation and immunodetection patterns obtained under test and basal conditions were compared, in order to identify the proteins selectively affected by the treatment with HNE. The protein spots detected by anti-HNE-histidine antibodies thus identified were manually excised from the gels with a clean scalpel, placed in Eppendorf tubes and subjected to in-gel digestion with trypsin, as detailed in Supplemental Materials and Methods. Matrix-assisted laser desorption ionization-time of flight/mass spectrometry (MALDI-TOF/MS) of the tryptic digests of HNE-immunoreactive protein spots was carried out with a PerSeptive Biosystems Voyager DE-PRO instrumentation (Framingham), equipped with a N_2_ laser (337 nm, 3 ns pulse width). Extraction solutions, containing tryptic digests from gel spots, were desalted and concentrated, using Poros Oligo R3 microcolumns. The columns were washed with 20 *μ*L of 0.1% TFA. Retained peptides were eluted directly onto the MALDI target, using 0.5 *μ*L of a matrix solution containing 10 mg of alpha-cyano-4-hydroxycinnamic acid (4-HCCA) in 1 mL of 50% aqueous acetonitrile. Mass spectra acquisitions were performed in positive reflectron mode, by accumulating 200 laser pulses. The accelerating voltage was 20 kV. External mass calibration was performed with low-mass peptide standards (PerSeptive Biosystems). Peptide assignments were accomplished using the GPMAW software (http://www.gpmaw.com/). MS data were searched against the NCBInr protein sequence data bases, using the Mascot server (http://www.matrixscience.com/).

### 2.12. Western Blot Analysis of HSP60 Expression after HNE Treatment of HL-60 Cells

In order to assess how treatment with HNE affected HSP60 expression, HL-60 cells were treated with 10 *μ*M HNE for 2 and 6 h, and 20 *μ*g of protein extracts was separated by SDS-PAGE and electroblotted onto nitrocellulose membranes (Bio-Rad Laboratories). These were blocked at 4°C overnight in Tris-buffered saline containing 5% milk, 0.5% Tween 20, and incubated at rt with the H-300 anti-HSP60 antibody (Santa Cruz Biotechnology) and then with a horseradish peroxidase-conjugated secondary antibody (Bio-Rad Laboratories). Detection was carried out by enhanced chemiluminescence (GE Healthcare). Densitometric gel analyses were performed either with the Multi-Analyst, version 1.1 software (Bio-Rad Laboratories), or the NIH ImageJ, version 1.48 software. All results were standardized using an anti-*β*-actin antibody.

### 2.13. Immunoprecipitation and Western Blot Analysis of HNE Adducts in HL-60 and THP-1 Cells Exposed to HNE or Native or Modified LDL

Total protein lysates (100–1000 *μ*g) of HL-60 cells were incubated with 2 *μ*g of H-300 primary anti-HSP60 antibody, under shaking at 4°C overnight. Thereafter, 35 *μ*L of protein A-Sepharose (Sigma Aldrich) was added at 4°C for 2 h. After centrifugation, the pellets were washed extensively in 0.02 M Tris/HCl, pH 7.4, 0.15 M NaCl, 1% Nonidet, 0.005 M EDTA, 0.001 M sodium orthovanadate, 0.001 M PMSF, and 0.05% aprotinin, mixed with 35 *μ*L of 0.2 M Tris/HCl buffer, pH 6.8, 2% SDS, 30% glycerol, and 16% 2-mercaptoethanol, and heated in boiling water bath for 2 min. Supernatants were subjected to SDS-PAGE and western blotting with the anti-HNE-histidine antibody, under the conditions already described for the immunodetection of cell proteins separated in 2-DE gels.

Total protein lysates (1000–1500 *μ*g) of THP-1 cells differentiated with PMA were subjected to preclearing with 25 *μ*L of a 1 : 1 mix of protein A-Sepharose 4B, Fast Flow (Sigma-P9424) and Protein G Sepharose 4B, Fast Flow (Sigma-P3296) at 4°C overnight, under rotary shaking. After centrifugation, the supernatants were incubated with 1 *μ*g per sample of the N-20 primary goat anti-HSP60 antibody at 4°C overnight, followed by 30 *μ*L of 1 : 1 Protein A : Protein G-Sepharose mix at 4°C for 2 h. After four washes in lysis buffer (0.02 M Tris/HCl, pH 7.4, 0.15 M NaCl, 1% Triton X-100, 10% glycerol, plus protease and phophatase inhibitors), immunoprecipitated proteins were detached from protein A + G by the addition of 12 *μ*L of concentrated Laemmli sample buffer and incubation at 60°C for 2 h. Immunoprecipitates were separated by SDS-PAGE in 8% acrylammide minigels and transferred to PVDF membranes in 0.025 M Tris, 0.192 M glycine, pH 8.3, and 20% methanol. The membranes were blocked with 10% NDM in PBS, pH 7.4, 0.1% Tween 20 (PBS-Tween) at rt for 1 h, rinsed trice in PBS-Tween and incubated with the anti-HNE-histidine primary antibody, at the dilution of 1 : 2000 in 5% NDM in PBS-Tween, at 4°C overnight. After three washes of 5 min each in PBS-Tween, the membranes were incubated with HRP-conjugated, secondary anti-mouse IgG antibodies, at the dilution of 1 : 5000 in 2.5% NDM in PBS-Tween, at rt for 1 h. After three washes in PBS-Tween, chemiluminescence was detected using the ECL Western Blotting Reagent (GE Healthcare).

### 2.14. Statistical Analysis

The identifications of HNE-protein adducts, obtained by comparing the protein sequences in the NCBInr database with the sequences determined by the MALDI-TOF-MS analyses of the products of the in-gel digestions of the HNE-immunoreactive spots from the 2-DE separations of the proteome of HL-60 cells treated with HNE, were probed by submitting queries to the MASCOT (Matrix Science) interface. The latter calculated the statistical significance of identifications (expressed both in the form of the minimal score corresponding to a statistical significance *P* < 0.05 and in the form of the expectancy of random identity), on the base of the following parameters: (1) the number of peptides whose sequences matched the protein sequence in the NCBInr database, divided by the number of sequenced peptides; (2) the percent fraction of the sequence of the identified protein covered by the sequenced peptides.

## 3. Results

### 3.1. Identification of Adducts of HNE within the Proteome of HL-60 Cells

**Figure 1:**
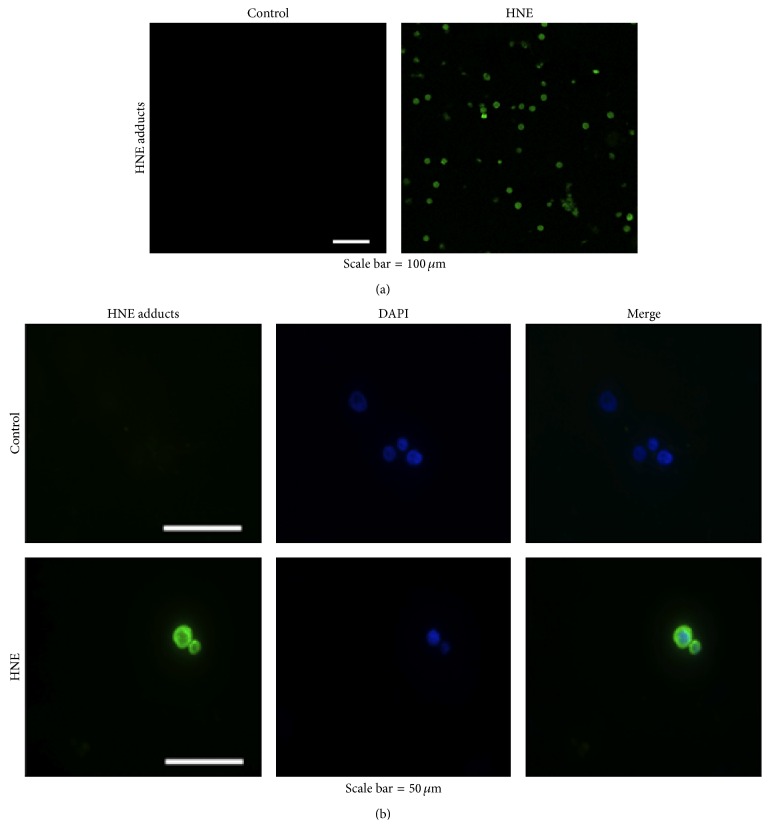
Confocal and epifluorescence microscopic analysis of HL-60 cells treated with 10 *μ*M HNE for 2 h. HL-60 cells (5 × 10^4^) were harvested after 2 h of treatment with 10 *μ*M HNE, fixed, permeabilized, blocked, and incubated overnight with the anti-HNE-histidine primary antibody at 4°C, as described in Section 2. Cells were then washed and exposed to the FITC-conjugated secondary antibody for 1 h at rt, washed again, mounted on coverslips, and examined under a Leica TCS SP2 confocal microscope, equipped with a water immersion HCX Apo 0.8 objective (a). For epifluorescence microscope analysis (Axioskop, Carl Zeiss), slides were also counterstained with DAPI (b).

**Figure 2:**
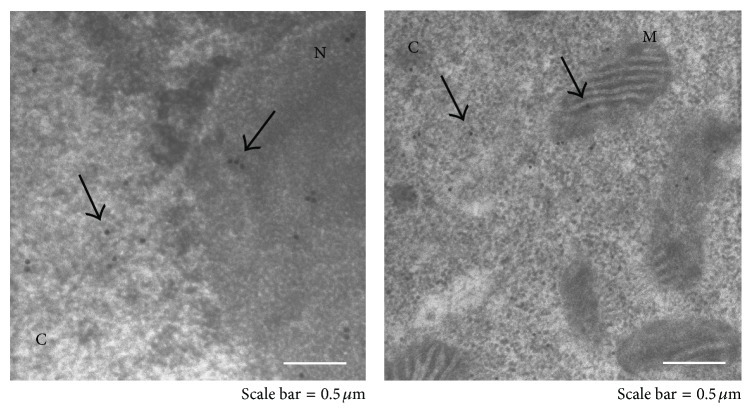
Electron microscopic analysis of HL-60 cells treated with 10 *μ*M HNE for 2 h. HNE-treated HL-60 cells were exposed to the anti-HNE-histidine antibody and subjected to electron microscopy and single-antibody immunogold localization as described in Section 2. Black arrows mark immune complexes between HNE-modified proteins and anti-HNE-histidine antibodies in cytosol (C), nuclei (N), and mitochondria (M).

The present investigation followed a previous study of ours [10], in which we showed that, in HL-60 cells treated with nontoxic doses (10 *μ*M) of 4-hydroxy-2-nonenal for 15 min, HNE immunoreactivity was principally localized at the level of the plasmamembrane and adducts of HNE were formed with the membrane-associated isoform of *α*-enolase, whereas in cells subjected to a longer-lasting treatment (2 h), HNE immunoreactivity diffused through the cytoplasm and some of it was detected also in the nuclei. In the present study, we sought to identify other targets of HNE adduct formation in HL-60 cells treated with 10 *μ*M HNE for 2 h. In preliminary experiments using confocal, epifluorescence, and electron microscopy, we confirmed that, after HNE being added to HL-60 cells, HNE completely pervaded them in 120 min. In treated cells, HNE immunoreactivity was uniformly diffused in the cytosol and the nuclei, while none was detected with the anti-HNE-histidine monoclonal antibody in control cells (Figures 1(a) and 1(b)). Electron microscopic analysis of the subcellular distribution of HNE, using a single-antibody immunogold localization technique, confirmed the cytosolic, mithocondrial, and nuclear localization of HNE adducts (Figure 2). Treatment with 10 *μ*M HNE did not exert either cytotoxic or prooxidant effects in HL-60 cells, as demonstrated by the trypan blue dye exclusion test and by DCFH-DA analysis (data not shown).

**Figure 3:**
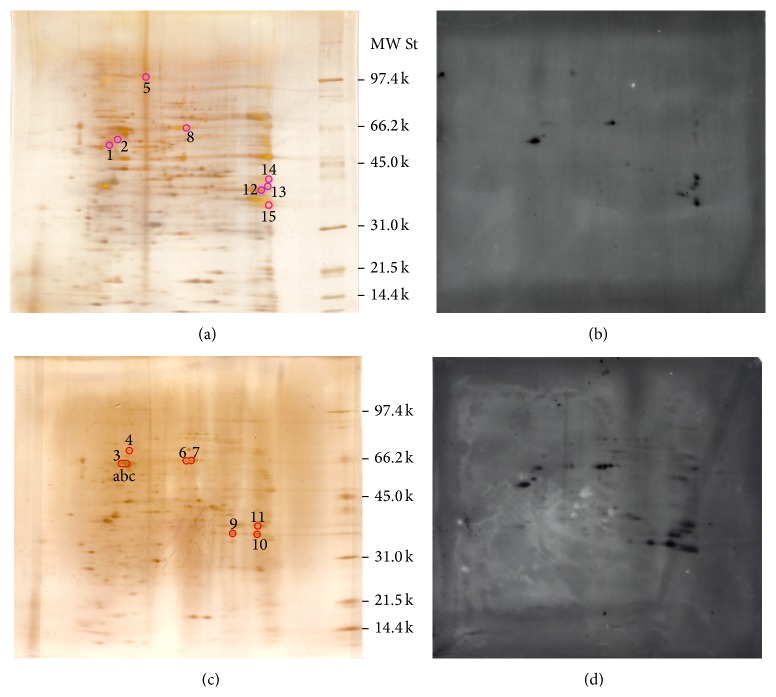
Separation by two-dimensional electrophoresis (2-DE) of the proteome of HL-60 cells under basal conditions ((a), (b)) and after exposure to 10 *μ*M HNE at 37°C for 2 h ((c), (d)). Total cell extracts, each containing from 50 to 200 *μ*g of total protein, were prepared from 1 × 10^6^ HL-60 cell aliquots. The gels in (a) and (c) were silver-stained, and those in (b) and (d) were electrophoretically transferred to PVDF membranes and probed with a murine anti-HNE-histidine monoclonal antibody, which was detected with a secondary anti-murine IgG antibody, with chemiluminescent technique. Molecular weight standards (MW St) in kDa are marked at right of the gels in (a) and (c). The signal detected by the use of the anti-HNE-histidine antibody in the immunoblots of (b) and (d) was used to identify, by visual alignment, the corresponding spots in the gels shown in (a) and (c). The spots thus identified were assigned numbers. The spots identified in the proteomes of both control and test cells are circled in purple, and those selectively identified in HNE-treated cells are circled in red.

**Figure 4:**
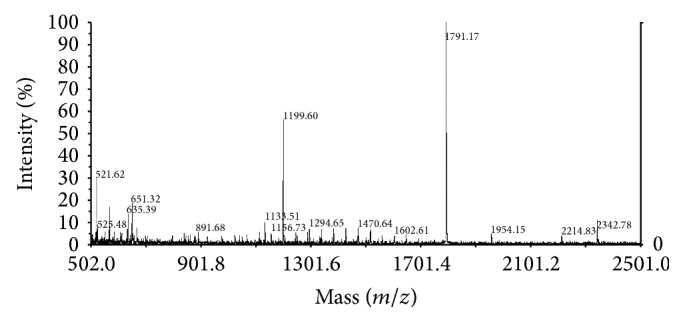
MALDI-TOF spectrum of the products of the in-gel tryptic digestion of spot number 3c from a preparative replica (200 *μ*g protein load) of the gel shown in Figure 3(c). MALDI-TOF-MS was performed as described under Section 2. Numbers besides mass signals indicate the mass-to-charge ratios (*m*/*z*) of the multiple pseudo-molecular ions ([M^+^H^+^]) deriving from the ionization of tryptic peptides.

**Figure 5:**
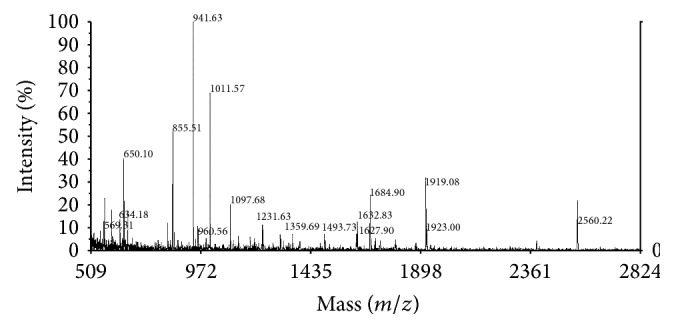
MALDI-TOF spectrum of the products of the in-gel tryptic digestion of spot number 4 from a preparative replica (200 *μ*g protein load) of the gel shown in Figure 3(c). MALDI-TOF-MS was performed as described under Section 2. Numbers besides mass signals indicate the mass-to-charge ratios (*m*/*z*) of the multiple pseudo-molecular ions ([M^+^H^+^]) deriving from the ionization of tryptic peptides.

Protein extracts from control and treated cells were prepared after treatment with HNE at 37°C for 2 h for separation by 2-DE and analysis.  Figure 3 shows silver-stained 2-DE polyacrylamide gels of total cell proteins of control (Figure 3(a)) and HNE-treated HL-60 cells (Figure 3(c)) and the respective immunoblots, prepared by electrophoretic transfer of the respective gel replicas onto PVDF and probing with a murine monoclonal anti-HNE-histidine antibody (Figures 3(b) and 3(d)). The spots marked as numbers 1, 2, 5, 8, 12, 13, and 14 in the gels shown in Figures 3(a) and 3(c) were not further analyzed, as they were present in both control and test cells. Since HNE was not detectable in control cells, these spots probably reflected some unspecific reactivity of the anti-HNE-histidine antibody. On the contrary, the spots numbers 3, 4, 6, 7, 9, and 10, obtained from 200 *μ*g of total protein extracts of HL-60 cells treated with 10 *μ*M HNE at 37°C for 2 h, were subjected to in-gel tryptic digestion, as described under Section 2, and analyzed by MALDI-TOF/MS. The peptide masses obtained from the MALDI-TOF/MS spectra of the tryptic digests of spots number 3c and 4 were aligned with the NCBInr data base, using the Mascot server. The analysis of spot number 3c identified 17 peptides (Figure 4), 9 of which had experimentally determined masses matching the theoretical masses of tryptic peptides (deriving from the cleavage at the carboxyl side of Lys or Arg residues, unless the next residue was Pro) of the human mitochondrial isoform of heat shock 60 kDa protein 1 (chaperonin) (NCBI id: gi|41399283|ref|NM_002156.4|; Uniprot identifier: P10809), with a theoretical M_*r*_ of 61187 and a nominal pI of 5.70. The MASCOT interface (Matrix Science) assigned to this identification the score 103 (to be compared to a threshold score of 56 for a statistical significance with *P* < 0.05) and an expectancy of 1e-06 (Supplemental Figure S1). The analysis of spot 4 identified 15 peptides (Figure 5), 12 of which had experimentally determined masses matching the theoretical masses of tryptic peptides of the putative uncharacterized protein CXorf49 (NCBI id: gi|223468692|ref|NM_001145140.1|; Uniprot identifier: A8MYA2), with a calculated M_*r*_ of 54611 and a nominal pI of 9.26. The MASCOT interface (Matrix Science) assigned to this identification the score 161 (to be compared to a threshold score of 67 for a statistical significance with *P* < 0.05) and an expectancy of 2.1e-11 (Supplemental Figure S2).

### 3.2. Previous Mass Spectrometric Identifications of Uncharacterized Protein CXorf49 in the PRIDE Proteomic Database

A search for A8MYA2 in the EMBL-EBI PRIDE Archive-proteomics data repository (http://www.ebi.ac.uk/pride/archive/) disclosed the following proteomic analyses and mapping projects of tissues or cell types, in which mass spectrometry had previously revealed, among the peptides identified, one or more peptides belonging to the uncharacterized protein CXorf49:

1. the extracellular matrix of human aorta (PRIDE id: PRD000269) and of human healthy versus aneurysmatic abdominal aorta (PRIDE id: PRD000416-417) [11];
2. human plasma (PRIDE id: PXD000605);
3. human cerebro-spinal fluid (PRIDE id: PXD000651-657);
4. the human oligodendrocyte-derived MO3.13 cell line (PRIDE identifier PXD000263) [12];
5. skinned rat cardiomyocyte myofilaments (PRIDE id: PRD000165) [13];
6. AKT2-interacting proteins in HEK293T cells (PRIDE id: PXD000197);
7. proteins interacting with the histone deacetylase inhibitor suberoylanilide hydroxamic acid (PRIDE id: PRD000443).

### 3.3. Expression of HSP60 in Control and HNE-Treated HL-60 cells

**Figure 6:**
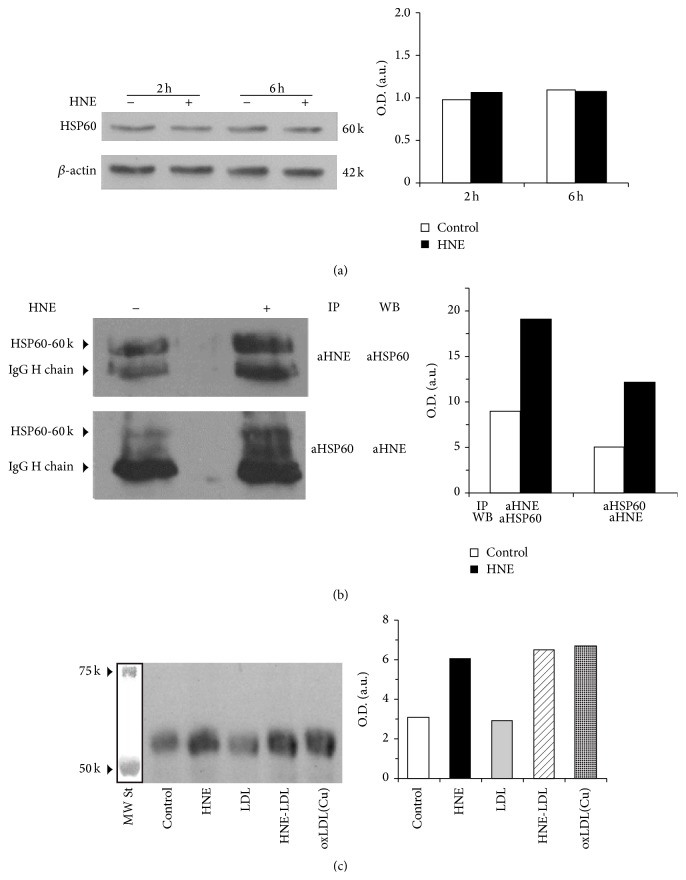
Immunoprecipitation and western blot analysis of HSP60 expression and of HNE-HSP60 adduct formation in HL-60 and THP-1 cells treated with HNE or with native or modified LDL. (a) left: lanes 1 and 3, control HL-60 cells incubated without additions for 2 and 6 h, respectively; lanes 2 and 4, HL-60 cells exposed to 10 *μ*M HNE for 2 and 6 h, respectively. *β*-actin was used as a quantitative reference. (b) left: immunoprecipitation of HL-60 cell protein extracts with anti-HNE-histidine antibodies (aHNE), followed by western blot with anti-HSP60 antibodies (aHSP60) (above); immunoprecipitation of HL-60 cell protein extracts with anti-HSP60 antibodies (aHSP60), followed by western blot with anti-HNE-histidine antibodies (aHNE) (below). (c) left: western blot with anti-HNE-histidine antibodies of the protein extracts subjected to immunoprecipitation with N-20 anti-HSP60 antibodies, from THP-1 cells differentiated with PMA and exposed for 4 h to 10 *μ*M HNE (HNE), 20 *μ*g/mL of native LDL (LDL), 20 *μ*g/mL of LDL modified with 1 mM HNE (HNE-LDL) at 37°C for 20 h, or 15 *μ*g/mL of oxLDL(Cu), that is, LDL modified by metal-catalyzed oxidation with 20 *μ*M Cu(SO_4_) at 37°C for 20 h. The positions of the molecular weight standards (MWSt) of 50 and 75 kDa are marked at left. All panels, at right: densitometric scans, in the form of histograms, of the HSP60- and HNE-immunoreactive bands of the respective immunoblots at the left side of the panels.

In order to determine how the treatment with HNE possibly affected the expression of HSP60 in HL-60 cells, we performed western blots for HSP60 in control and HNE-treated HL-60 cells. HSP60 expression was not modified after the addition of 10 *μ*M HNE to test cells (Figure 6(a)).

### 3.4. Immunoprecipitation and Western Blot Analysis of HNE-HSP60 Adducts in HL-60 and THP-1 Cells

In order to confirm the formation of HNE adducts with HSP60, we performed immunoprecipitations of the HL-60 cell lysates obtained under basal conditions and after 2 h of exposure to 10 *μ*M HNE. Crossed immunoprecipitation with anti-HNE-histidine antibodies, followed by western blotting with anti-HSP60 antibodies, and immunoprecipitation with anti-HSP60 antibodies, followed by western blotting with anti-HNE-histidine antibodies, confirmed the formation of the HNE-HSP60 adducts (Figure 6(b)). Limited amounts of HNE- and HSP60-cross-reactive material were detected by this combined approach also in control cells. However, the amounts of HNE-HSP60 adducts formed in stimulated HL-60 cells were well above those evidenced under basal conditions.

We next addressed the question, by a similar immunoprecipitation and western blotting strategy, whether HNE in protein-bound form, particularly HNE bound to apolipoproteins in oxidized LDL, a well-known inducer of macrophage and endothelial dysfunction [21–30], might incite in target cells the production of endogenous HNE and the formation of adducts of HNE with cell proteins, particularly HSP60. To this aim, we decided to test the human monocytic leukemic THP-1 cell line. These cells have been reported to represent, after differentiation induced with phorbol 12-myristate 13-acetate (PMA), a useful model, for experimental purposes, of the interactions of human monocytes-macrophages with vascular cells during vascular inflammation, particularly atherogenesis [36]. LDL were isolated from a healthy, normolipemic donor, under conditions minimizing spontaneous oxidation [37], to serve as a negative control. Moreover, we modified LDL both by direct reaction with HNE (1 mM at 37°C for 20 h) and by metal-catalyzed oxidation with CuSO_4_ (20 *μ*M at 37°C for 20 h). The latter was proven to produce various oxidative modifications of LDL, including the formation of HNE-histidine adducts [41]. Differentiated THP-1 cells were tested also by exposure to 10 *μ*M HNE, as a positive control. Before the addition of stimuli, the cells were cultured for 10 h in RPMI 1640 medium without FBS, in order to avoid the confounding effects of LDL from fetal bovine serum. Stimulation was carried out for 4 h, since it was reported that, in differentiated THP-1 cells exposed to 10 *μ*M HNE or to carbon nanoparticles, accumulation of HNE adducts with cell components was seen starting from 4 h after the addition of stimuli on [42]. The results are shown in Figure 6(c). In THP-1 cells differentiated with PMA and exposed to HNE, HNE-LDL, or oxLDL(Cu) (LDL oxidized with CuSO_4_), but not to freshly isolated, native LDL, HNE adducts with HSP60 were formed at levels distinctly higher than under basal conditions.

## 4. Discussion

The experiments reported here led us to identify HSP60 and uncharacterized protein CXorf49 out of a small group of components of the HL-60 cell proteome that were most prone to form HNE adducts, upon exposure to nontoxic (10 *μ*M) HNE concentrations. The human promyelocytic leukemic line of HL-60 cells was selected in view of its lack of endogenous peroxydizing ability, while the human monocytic leukemic line of THP-1 cells was used, after differentiation induced with PMA, as a model of the macrophagic responses to cell stressors, such as oxLDL [36]. A number of recent redox proteomic approaches, mainly in the field of neurodegenerative diseases, have pinpointed the susceptibility of defined subsets of cell proteins, including enzymes of metabolism, oxidative phosphorylation, and stress response proteins, to modification by lipid peroxidation products [7, 8]. The susceptibility of HSP60 evidenced in our study may reflect its participation in the maintenance of proteins exposed to oxidative damage, at cellular sites of increased production of reactive oxygen species (ROS).

Human HSP60 is a member of a superfamily of highly conserved chaperones, whose synthesis, exposure at the cell surface, and extracellular release are upregulated in response to stressing conditions. By associating with endogenous cellular peptides, HSPs also allow their efficient presentation to T cells. Dying cells release HSPs complexed with cell antigens that are internalized via cell-surface receptors by APCs, thereby facilitating antigen transfer and presentation [43]. The binding and internalization of HSP60 are mediated by the scavenger receptor lectin-like oxidized LDL receptor-1 (LOX-1), which is also the main receptor of proatherogenic oxidized LDL (oxLDL) at the surface of ECs, monocyte-derived macrophages, and vascular smooth muscle cells (SMCs) [26]. HSP60 and other HSPs are also powerful danger signals (DAMPs), inducing the TLR-4-dependent maturation and activation of APCs [18, 44]. Despite prevailing evidence in favor of their proinflammatory actions [16], extracellularly released HSPs display immunomodulatory suppressive effects as well [45, 46]. Factors influencing this balance include the level of HSP expression and the molecular form in which HSPs are released from cells in connection with the modality of cell death. High levels of HSPs passively released from necrotic cells are immunostimulatory, while HSPs secreted in exosome-bound form by apoptotic cells are immunosuppressive [47, 48].

The data that we report stimulate a reflection on the possible impact that the modification of HSP60 with HNE might have in relation to its involment in the pathogenesis of human disease, particularly atherosclerosis. Associations were reported between high levels of HSP60 in plasma and early carotid atherosclerosis [49, 50] and coronary heart disease [51]. In patients with angiographycally normal coronary arteries, the serum concentration of HSP60 correlated with the extent of cardiac and microvascular dysfunction [52]. Moreover, the presence of HSP60-reactive T cells in atherosclerotic lesions, especially in the early phases of disease, was repeatedly documented [30, 32–35, 53–56].

Atherosclerosis can be viewed as an immunoinflammatory condition, in which a persisting inflammatory stimulus sets the conditions for the breaking of tolerance to self-antigens, thus initiating an adaptive autoimmune response, which, in turn, perpetuates the inflammatory process. Early lesions are first infiltrated by activated T cells [54, 57, 58], which join the network of vascular-associated dendritic cells (VADCs) [59] followed by monocytes/macrophages and vascular SMCs, which predominate in advanced atheromatous plaques [54, 57, 58]. The concept of atherosclerosis as an autoimmune response against HSP60, proposed in 1992 [60], has gained support in time [30, 61]. In this model, all known risk factors set the stage for the immunoinflammatory process, by acting as EC stressors sharing the ability to induce the surface expression of adhesion molecules and HSP-60. The former foster the influx of oxLDL and mononuclear cells into the arterial intima, and the latter may function as a DAMP, aiding in the triggering of autoreactive T-cells, and a self-antigenic target.

Investigating the possible consequences of the modification of HSP60 with HNE seems warranted, given the proatherogenic role played in atherosclerosis of humans [24, 25] and* LOX-1-null* mice [29] by oxLDL, which shares with HSP60 the LOX-1 receptor. LOX-1 is upregulated in ECs upon exposure to oxLDL [23]. Upon binding to LOX-1, oxLDL induced the expression of adhesion molecules [62] and monocyte chemoattractant protein-1 (MCP-1) [24] and promoted the production of reactive oxygen species (ROS), NF-*κ*B activation [25, 27, 63], and apoptosis [23], all of which characterized the endothelial dysfunction, a crucial early step in atherosclerosis [22, 26]. The formation of HNE adducts with apolipoprotein B (Apo B) in LDL was strongly implicated in the proatherogenic properties of oxLDL, as it converted LDL into an atherogenic form taken up by macrophages, leading to the formation of foam cells [64]. In competition binding experiments, HNE-histidine adducts served as LOX-1 ligands in oxLDL, HNE-LDL, and HNE-BSA [41]. The binding stimulated ROS formation and activated a pathway of MAPK and NF-*κ*B activation [21].

There are several reasons of interest in finding HNE-HSP60 adducts in HL-60 cells exposed to HNE and, to an even greater extent, in THP-1 cells exposed to HNE and HNE-modified LDL:

(1) The finding that HSP60 is also a target of the lipoxidative damage that renders oxLDL proatherogenic and that HNE-LDL themselves can act as a trigger not only for the upregulation of HSP60 expression [30, 65], but also for its modification with HNE, sets a scenario, in which different HNE targets (LDL, HSP60), exerting diverse effects at a common receptor site (LOX-1), might contribute synergystically to oxidative stress-induced inflammation. In addition, due to its ability to convey endogenous antigens into the presentation machinery of APCs, HNE-HSP60 might favour the lipoxidative modification of other proteins, one example of which may be uncharacterized protein CXorf49, thus enforcing a “lipoxidative spread.”

(2) The modification of HSP60 with HNE might affect its interactions with innate receptors at the surface of ECs, macrophages, VADCs, and their consequences on the balance between tolerizing and positive costimuli provided by APCs to autoreactive T cells in conjunction with self-antigenic determinants [66]. In this regard, it is worth noticing that the binding of oxLDL to LOX-1 at the surface of DCs induced the upregulation of scavenger receptors and the maturation and differentiation of DCs, with the expression of costimulatory molecules [31].

(3) Furthermore, the formation of HNE adducts might be the modification of self that is still required for the breaking of immunological tolerance to HSP60 and the triggering of adaptive autoimmunity. Infections with* Chlamidia pneumoniae* have been indicated as a risk factor of atherosclerosis. The susceptibility to vascular disease would be the price paid by human beings for developing protective immunity against microbial infections, due to the high degree of sequence homology between bacterial HSP60's (over 95%) and between prokaryotic and mammalian HSP60 (over 50%) [30, 61]. However, under physiological conditions, humans seem to be tolerant to autologous HSP60, with negative central selection [67, 68] and peripheral failsafe mechanisms preventing the onset of autoimmunity against HSP60 [69]. The associations reported between the occurrence and severity of cardiovascular disease and the titers and cross-reactivities of antibodies against human and bacterial HSP60's were inconsistent (reviewed in [30]). On the other hand, epitopic mimicry between bacterial and human HSP60 may not be the sole way of triggering an autoimmune response. Modification of human HSP60 with HNE as a result of lipid peroxidative damage might be just another way.

It is worthy noting that, in a recent report [70], the ability of LDL modified by oxidation or by human group X-secreted phospholipase A2 to induce DC activation and Th1 and Th17 cell differentiation was attenuated by lentiviral-mediated shRNA knockdown of HSP60 and HSP90 in DC, which indicates their involvement in the activation of T-cell responses in atherosclerosis.

Ultimately, the modification of HSP60 with HNE might both contribute to the oxidative stress-driven inflammation of the arterial intima and act as a switchover to the immunity-driven perpetuation of the inflammatory disease process. Verification of these hypotheses will entail gaining proof for the occurrence of the modification of HSP-60 with HNE* in vivo* and studying its functional consequences on DCs and human ECs. The results may confirm or rule out the significance of HNE-modified HSP60 as a marker/predictor of atherosclerosis.

## 5. Conclusions

In the human promyelocytic leukemic HL-60 cell line exposed to a nontoxic concentration (10 *μ*M) of HNE, HSP60 alongside uncharacterized protein CXorf49 was among the cell proteins most susceptible to the formation of HNE adducts. In human monocytic leukemic THP-1 cells differentiated with PMA the formation of HNE adducts with HSP60 was confirmed upon exposure to HNE, but also in response to LDL modified with HNE or by copper-catalyzed oxidation, but not to native LDL.

In the light of the well-established pathogenic role of HSP60 as a target of autoimmune adaptive responses in atherosclerosis, the interest of this observation is severalfold.

(1) Since HSP60 shares the scavenger receptor LOX-1 with oxidized LDL, a determinant of EC dysfunction and foam cell formation in early stages of atheroma formation, and because HNE-histidine adducts are a major determinant of oxLDL binding to LOX-1, it is probable that HNE-HSP60 may act synergistically with oxLDL and other EC stressors.

(2) As the fate of the presentation of HSP-associated antigens is affected by the inflammatory milieu, the mode of cell death, and the molecular form and mechanism of extracellular release of HSPs, HNE modification might affect the proven ability of HSP60 to induce DC maturation and differentiation into a T-effector-, rather than a T-regulatory-promoting phenotype.

(3) The modification of HSP60 with HNE might represent the necessary self-modification to attain the breaking of immunological tolerance, and this, in turn, may herald the transition of the atherosclerotic process from oxidative stress-driven to immunity-driven chronic inflammation.

## Supplementary Material

Figure S1: Print-out of the query to the MASCOT interface (Matrix Science), concerning the mass spectrum (shown in Fig. 3) of the in-gel tryptic digestion products of spot number 3c from a preparative replica (200 *μ*g protein load) of the gel shown in Fig. 2.
Figure S2: Print-out of the query to the MASCOT interface (Matrix Science), concerning the mass spectrum (shown in Fig. 4) of the in-gel tryptic digestion products of spot number 4 from a preparative replica (200 *μ*g protein load) of the gel shown in Fig. 2.

## Abbreviations

DC: Dendritic cell
DCFA-DA: 2′-7′-Dichlorofluorescein diacetate
EC: Endothelial cell
FBS: Fetal bovine serum
4-HCCA: Alpha-cyano-4-hydroxycinnamic acid
HNE: 4-Hydroxy-2-nonenal
HSP60: Heat shock 60 kDa protein 1
LDL: Low-density lipoprotein
LOX-1: Lectin-like oxidized LDL receptor-1
LRW: London Resin White
NDM: Nonfat dry milk
oxLDL: Oxidized LDL
PMA: Phorbol 12-myristate 13-acetate
PMSF: Phenylmethanesulfonyl fluoride
PVDF: Polyvinylidene difluoride
ROS: Reactive oxygen species
SMC: Smooth muscle cell
VADC: Vascular-associated dendritic cell.
