# Supplementary material for: Generation of Adducts of 4-Hydroxy-2-nonenal with Heat Shock 60 kDa Protein 1 in Human Promyelocytic HL-60 and Monocytic THP-1 Cell Lines
Source: Oxid Med Cell Longev. 2015 May 20;2015:296146. doi: 10.1155/2015/296146 (PMC4452872; doi:10.1155/2015/296146)
Supplement: Supplementary file 1 — Figure S1: Print-out of the query to the MASCOT interface (Matrix Science), concerning the mass spectrum (shown in Fig. 3) of the in-gel tryptic digestion products of spot number 3c from a preparative replica (200 μg protein load) of the gel shown in Fig. 2. Figure S2: Print-out of the query to the MASCOT interface (Matrix Science), concerning the mass spectrum (shown in Fig. 4) of the in-gel tryptic digestion products of spot number 4 from a preparative replica (200 μg protein load) of the gel shown in Fig. 2. [file 296146.f1.docx]

Manuscript: Generation of adducts of 4-hydroxy-2-nonenal with heat shock 60kDa protein 1 in human promyelocytic HL-60 and monocytic THP-1 cell lines.

A possible switchover from oxidative stress-driven to immunity-driven inflammation on the way to atherosclerosis

by Alessia Arcaro, Martina Daga, Giovanni Paolo Cetrangolo, Eric Ciamporcero, Alessio Lepore, Stefania Pizzimenti, Claudia Petrella, Maria Graf, Koji Uchida, Gianfranco Mamone, Pasquale Ferranti, Paul RJ Ames, Giuseppe Palumbo, Giuseppina Barrera and Fabrizio Gentile

**SUPPLEMENTAL DATA**

**SUPPLEMENTAL MATERIALS AND METHODS**

*Two-dimensional polyacrylamide gel electrophoresis (2-DE)* *–* IEF in immobilized, linear pH gradients was performed using up to four 11-cm-long, 0.1-mm-thick, pre-cast Immobiline DryStrips, pH 3-10L (GE Healthcare, Milan, Italy) at the same time, using a Multiphor II flatbed electrophoresis unit, equipped with an Immobiline DryStrip IEF kit, connected to a MultiTemp II thermostatic circulator (GE Healthcare, Milan). Samples were cup-loaded at intermediate distance between the anode and the cathode, onto Immobiline DryStrips, previously rehydrated in 300 μL of 8 M urea, 2% 3-[(3-cholamido-propyl)-dimethylammonium]-propansulfonate (CHAPS), 2% Immobilized pH Gradient Buffer (IPG Buffer, GE Healthcare), 2.8 mg/mL dithiothreitol, and bromophenol blue as tracking dye (rehydration buffer), for 16 h at room temperature. For preparative purposes, up to 100 μg of cell total proteins were dissolved in rehydration buffer, so that sample loading into the Immobiline DryStrips occurred at the same time as rehydration. IEF was conducted for 30 min at the constant voltage of 500 V, followed by 30 min 1500 V and 8 h at 3000 V at 15 °C, after which the proteins in the DryStrips were reduced and alkylated, by equilibration in 10 mL of 0.075 M Tris/HCl buffer, pH 8.8, containing 6 M urea, 2% SDS, 30% glycerol, trace amounts of bromphenol blue, plus 1% dithiothreitol for 15 min at rt, followed by another 15 min in 10 mL of the same buffer, in which dithiothreitol was replaced by 2.5% iodoacetamide. After this treatment, each Immobiline DryStrip was layered onto a 11-cm-long, 14-cm-wide, 1.5-mm-thick vertical gradient gel, containing 8-18% total acrylamide in 0.375 M Tris/HCl, pH 8.6, 0.1% SDS, and soldered to it with 4 mL of a 3.75% polyacrylamide gel in electrode buffer (0.025 M Tris base, 0.2 M glycine, 0.1% SDS). SDS-PAGE was conducted in two Hoefer SE600 vertical electrophoresis units, at the constant current of 10 mAmp per gel, for 16 h at 15 °C. Molecular mass standards (Bio-Rad Laboratories, Segrate, Italy) were: phosphorylase *b* (97,400), bovine serum albumin (66,200), ovalbumin (45,000), carbonic anhydrase (31,000), soybean trypsin inhibitor (21,500), lysozyme (14,400). At the end of the run, analytical gels were fixed overnight in 30% (vol/vol) ethanol, 5% (vol/vol) acetic acid for subsequent silver staining, the other replica was used for the immunodetection of HNE adducts.

*Electrophoretic transfer and immunodetection of proteins separated in 2-D gels* *–* The proteins separated by 2-DE were transferred from SDS-PAGE gels onto polyvinylidene difluoride

*Silver staining of SDS-PAGE gels* *–* Gel replicas run for analytical purposes were stained, using the PlusOne Silver Staining kit (GE Healthcare). Preparative gels destined for the identification of protein spots by mass spectrometry were stained using a modified, MS-compatible protocol. Briefly, gels were fixed overnight in 50% (vol/vol) methanol, 5% (vol/vol) acetic acid. After rinsing in 50% methanol for 10 min, and in milli-Q-purified (Millipore, Milan, Italy) water for another 10 min, the gels were sensitized with 0.2% sodium thiosulfate for 1 min, and then rinsed quickly twice again in milli-Q-purified water. The gels were then incubated in 0.1% silver nitrate for 20 min at 4 °C, after which they were rinsed in milli-Q-purified water twice more. Staining was developed in 0.04% formalin, 2% sodium carbonate, and blocked in 5% acetic acid. Gels were stained in 1% acetic acid at 4 °C until peaking of protein spots for MS analysis.

*In-gel tryptic digestion of HNE-immunoreactive spots from the 2-DE blots of the proteome of HL-60 cells treated with HNE* – The protein spots detected by anti-HNE-histidine-antibodies identified the 2-DE blots of the proteome of HL-60 cells treated with HNE were manually excised from the gels with a clean scalpel, placed in Eppendorf tubes and subjected to in-gel digestion with trypsin, as detaled in Supplemental Data. and washed twice with 50 μL of Milli-Q-purified water. Each gel piece was completely destained in 0.050 M NH_4_HCO_3_ in 50% (vol/vol) aqueous acetonitrile. Destained spots were dehydrated by submersion into acetonitrile, and dried under vacuum after acetonitrile removal. Each dried gel piece was incubated in 20 μL of 0.05 M NH_4_HCO_3_, containing 12 ng/μL of trypsin, on ice. After 45 min of digestion, the supernatant was removed and incubated overnight at 37 °C. The resulting tryptic digests were extracted in 40 μL of 50% acetonitrile, 2.5% formic acid and concentrated to a tenth of the original volume in vacuum centrifuge for Matrix-Assisted Laser Desorption Ionization-Time of Flight/Mass Spectrometry (MALDI-TOF/MS).

**SUPPLEMENTAL FIGURE LEGENDS**

**Figure S1.** *Print-out of the query to the MASCOT interface (Matrix Science), concerning the mass spectrum (shown in Fig. 3) of the in-gel tryptic digestion products of spot number 3c from a preparative replica (200 μg protein load) of the gel shown in Fig. 2.*

**Figure S2.** *Print-out of the query to the MASCOT interface (Matrix Science), concerning the mass spectrum (shown in Fig. 4) of the in-gel tryptic digestion products of spot number 4 from a preparative replica (200 μg protein load) of the gel shown in Fig. 2.*

**SUPPLEMENTAL FIGURES**

**
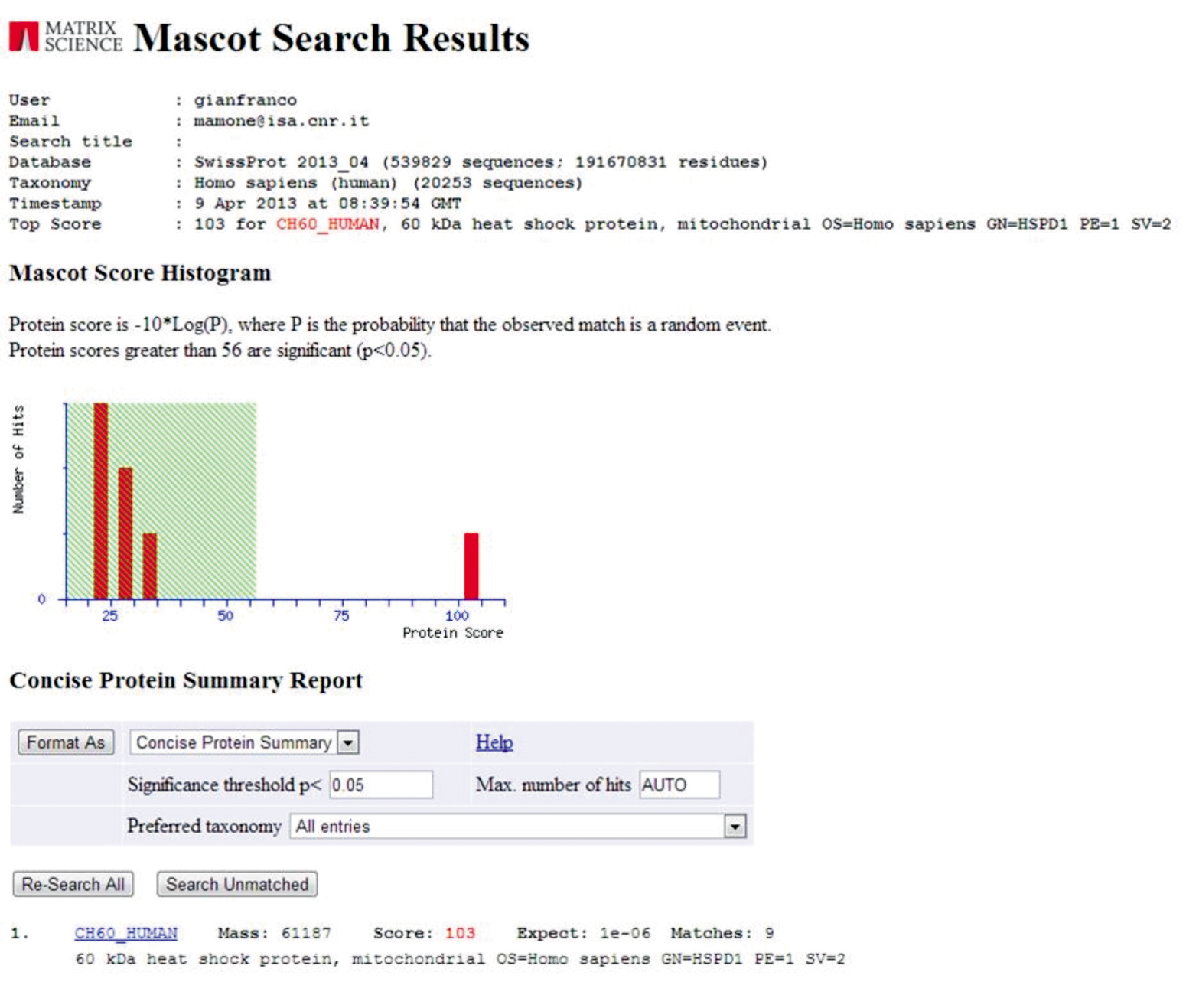
**

**Supplemental Figure S1**

**
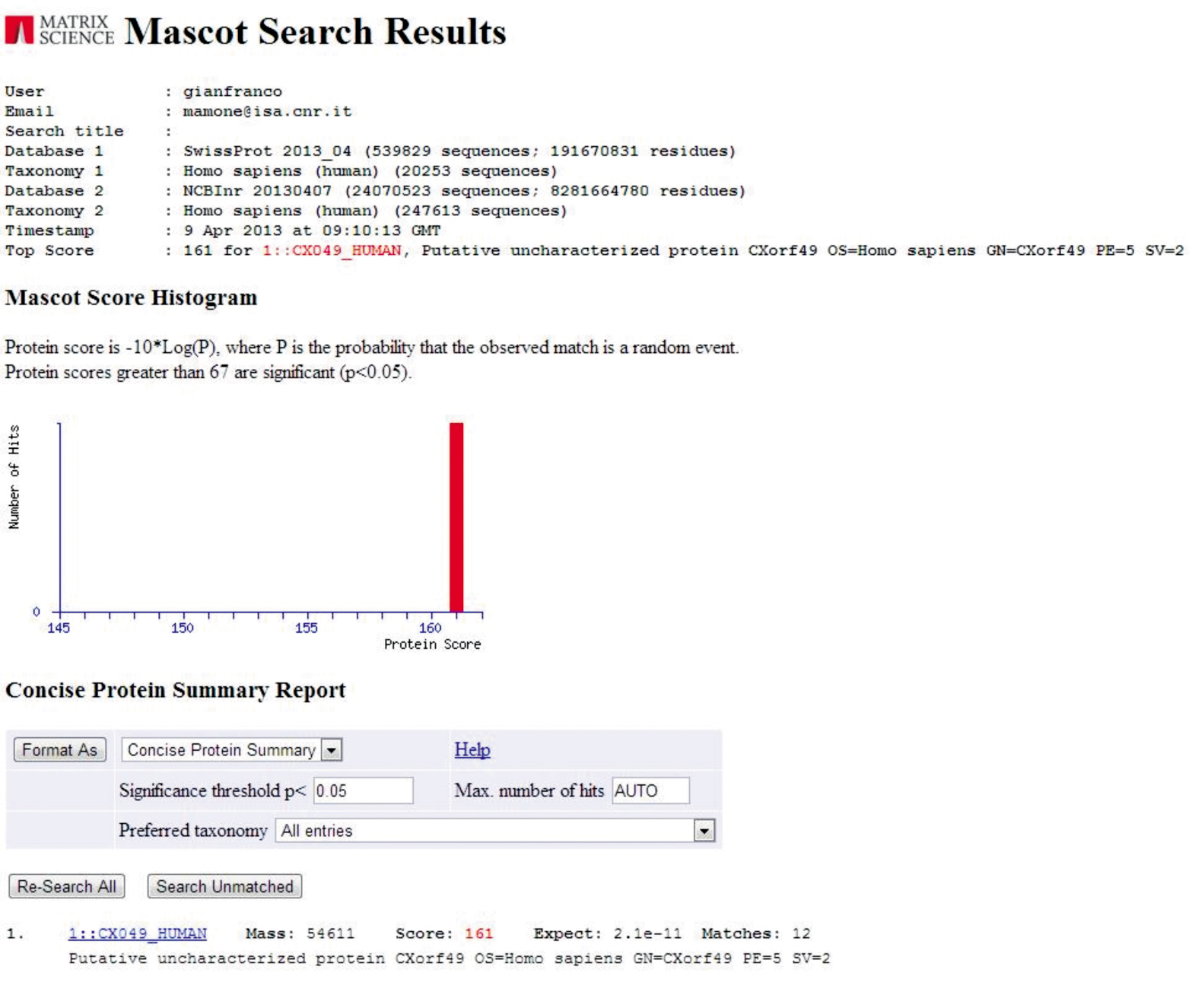
**

**Supplemental Figure S2**
